# Properties and Performance of Epoxy Resin/Boron Acid Composites

**DOI:** 10.3390/ma17092092

**Published:** 2024-04-29

**Authors:** Anna Rudawska, Mariaenrica Frigione, Antonella Sarcinella, Valentina Brunella, Ludovica Di Lorenzo, Ewa Olewnik-Kruszkowska

**Affiliations:** 1 Faculty of Mechanical Engineering, Lublin University of Technology, Nadbystrzycka 36, 20-618 Lublin, Poland; a.rudawska@pollub.pl; 2 Department of Innovation Engineering, University of Salento, Via Arnesano, 73100 Lecce, Italy; antonella.sarcinella@unisalento.it; 3 Department of Chemistry, University of Torino, Via P. Giuria 7, 10125 Torino, Italy; valentina.brunella@unito.it (V.B.); ludovica.dilorenzo@unito.it (L.D.L.); 4 Faculty of Chemistry, Nicolaus Copernicus University in Toruń, Gagarin 7 Street, 87-100 Toruń, Poland; olewnik@umk.pl

**Keywords:** boron acid, curing agents, epoxy resin composite, mechanical properties, thermal properties, flammability tests

## Abstract

This research study focused on the effect of adding boric acid to epoxy resin in order to obtain a composite material with improved properties and performance. To this end, a fine powder of boric acid (H_3_BO_3_) was introduced into epoxy resin in different amounts, i.e., 0.5 g, 1.0 g, and 1.5 g. As the matrix of the epoxy composites, styrene-modified epoxy resin based on bisphenol A (BPA) (Epidian 53) was used. It was cross-linked with two types of curing agents, i.e., an amine (ET) and a polyamide (PAC). The mechanical properties of the obtained epoxy composites (in terms of compressive strength, compressive modulus, and compressive strain) were determined at room temperature in order to assess the effect of the addition of boron acid and of the type of curing agent employed to cure the epoxy on these characteristics. Calorimetric measurements were made to highlight any changes in the glass transition temperature (Tg) as a result of the addition of boric acid to epoxy resin. Finally, flammability tests were performed on both Epidian 53/PAC and Epidian 53/ET epoxy composites to analyze their fire behavior and consequently establish the effectiveness of the selected additive as a flame retardant.

## 1. Introduction

Epoxy resins are thermosetting polymers [1,2,3,4] whose molecules contain at least two epoxy groups. They are extensively used in various fields, including adhesives, coatings, sealing materials, matrices for composite materials reinforced with different fibers/fillers, structural elements, laminates, and dielectric materials [5,6,7,8]. The most commonly used epoxy resins are those based on diglycidyl ether of bisphenol A (DGEBA) [4,9,10,11,12]. In order to obtain specific functional properties, epoxy resin is subjected to a curing (i.e., cross-linking) process in the presence of a suitable curing agent [6,13,14,15,16]. The selection of the most appropriate curing agent for the curing of an epoxy resin is one of the most important elements in the development of an epoxy material with the required functional characteristics [1,6,17,18]. As Gotto [19] emphasizes, “the correct choice of curing agent can dramatically improve the properties of the formulation such as heat resistance and flexibility while also allowing curing at lower temperatures for example”. It should be mentioned that the process of cross-linking of epoxy resins with various curing agents is widely discussed in the literature [17,20,21], but many authors emphasize that the mechanism of epoxy curing is complex [13,19].

In order to modify epoxy resins and create composites with optimal properties for a specific application, different compounds can also be chosen; they are added into the resin before the curing process takes place [3]. The epoxy resin and the selected curing agent thus form the matrix for the final composite [3,5]. An appropriate selection of components allows for improvements in terms of (i) mechanical, thermal, electrical, and dielectric properties [13,22,23,24], (ii) technological properties of materials that facilitate the processing, or (iii) providing special functional properties, e.g., low flammability, durability, low coefficient of friction, or resistance to dirt [25,26,27,28].

Among the properties mentioned, flame retardant properties are very important [4,29,30,31,32,33], and this applies to all polymeric materials, especially if used in particular sectors, such as in electrical works or in the construction field [26,34,35,36]. The presence of fragments derived from epichlorohydrin in the epoxy chain results in epoxy resins being characterized by a certain reduction in flammability compared to other halogen-free polymer materials. Unfortunately, this level of flame retardancy is insufficient in industrial practice, and these resins do not meet the increasingly stringent safety regulations [37].

In order to obtain better flame retardant properties, additives—antypirenes—with various chemical bases are used [4,30,35,38]. These include boron acid [25,39,40,41,42], phosphorus compounds [9,43], halogenated flame retardants (bromine and chlorine) [39], and halogen-free retardant [8,44], among others. Kandola et al. [44] reviewed the different types of flame retardants required to achieve a specific level of flame retardancy. Hamciuc et al. [25] presented a study focused on epoxy-based composites with improved flame-resistant properties, which were obtained by adding two flame retardant additives to the epoxy resin, one of them being boric acid. These authors noticed, inter alia, that the higher charring efficiency of epoxy-based composites containing H_3_BO_3_ was favorable for improving flame resistance. Nazarenko et al. [39] demonstrated that the incorporation of boric acid into the polymer matrix increases the thermal stability of epoxy composites and leads to a 2–2.7 times reduction in toxic gaseous products. Visakh et al. [42] showed that the thermal properties of the tested epoxy composites depend on the filler content. The results showed that the addition of 10% wt. fillers of both boric acid and natural zeolite significantly improved the thermal properties of the obtained composites. Flame retardants for epoxy resins can also contain combinations of elements that allow synergistic and cooperative effects to be achieved. For example, phosphorus–nitrogen-based modifiers and flame retardants of epoxy resins are presented by, e.g., Konstantinova et al. [45], Orlov et al. [46], Terekhov et al. [47], or Benin et al. [48].

In most of the cited works [25,32,36,42], however, the research was mainly focused on determining flame retardant properties [8], which are an extremely important aspect, especially when epoxies are used in building applications as resins for injection or for wall or floor coatings. Boric acid is a common, fairly economic additive that can act as an effective flame retardant for epoxy resins in such applications; it was, therefore, selected in this study. However, in the present work, attention has also been paid to mechanical and thermal properties. When designing structures containing composite materials, mechanical properties and thermal characteristics are as important as behavior in the presence of fire. In fact, both specific functional and mechanical properties [26,33,49] should be taken into account when selecting the most appropriate material for a specific application. Thermal properties, especially the glass transition temperature, determine the temperature range of use of resins and influence their durability. Due to the large number of epoxy resin types, curing agents, and modifying additives [3], as well as the numerous possibilities of mixing them in different quantitative compositions, investigations on these materials are still an interesting research field. In this work, therefore, the results of a research study focused on determining the effect of the addition of boric acid on the mechanical and thermal characteristics of an epoxy resin are presented. Fine-grained boric acid (H_3_BO_3_) powder was introduced into epoxy composites. A styrene-modified epoxy resin based on bisphenol A (BPA) was used as the matrix of the epoxy composites. The epoxy matrix was cross-linked with two types of curing agents, i.e., an amine and a polyamide. The effect of high temperatures as a function of the formulation of the epoxy composites was assessed by thermogravimetric analysis. To the best of the authors’ knowledge, the thermal and mechanical properties of epoxy composites containing boric acid have never been contemporarily analyzed in the literature. Finally, flammability tests were performed on unmodified epoxies and on those modified with boric acid.

## 2. Materials and Methods

### 2.1. Boron Acid/Epoxy Resin Composites

**Figure 1:**
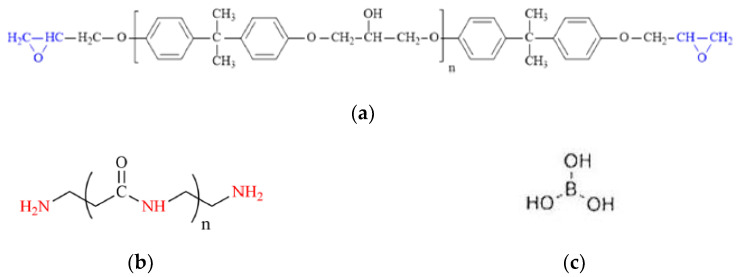
Schemes of (**a**) bisphenolic epoxy resin [56]; (**b**) polyaminoamide curing agent [56]; (**c**) ortho form of boric acid [48].

Boron acid/epoxy resin composites were produced using an epoxy resin based on bisphenol A (BPA), which is a mixture of a resin with an epoxy number of 0.48–0.51 mol/100 g with styrene solvent. Its scheme is presented in Figure 1a. The epoxy value of this epoxy resin (trade name Epidian 53, Sarzyna Resins, Nowa Sarzyna, Poland) is min. 0.41 mol/100 g; the viscosity range is 900–1500 m·Pas; the density at a temperature of 25 °C is 1.15 g/cm^3^; and the average molecular weight is ≤700. Amine (whose chemical structure is illustrated in Figure 1b) and polyamide curing agents were used to cure the epoxy resin. The properties of these curing agents are presented in Table 1 [50,51]. These agents are, in fact, often used to cure epoxy systems used as adhesives, coating materials, or other structural elements. In particular, the amine curing agent is mainly used for epoxies applied as a floor coating. In the case of structural adhesive applications, a polyamide is often used as the epoxy resin curing agent.

The epoxy resin/curing agent ratio resulted from the stoichiometric ratios of the epoxy resin and the curing agents, as specified in Table 1. The epoxy composites were produced by adding fine powder of boric acid (H_3_BO_3_, Chempur Company, Piekary Śląskie, Poland) to the resin. This compound has a wide range of applications [31,36,52,53,54], one of which is to improve the flame retardancy of polymers [31,39]. The ortho form of boric acid was used, as shown in Figure 1c [52]. Three amounts of boric acid were used, i.e., 0.5%, 1.0%, and 1.5% (per 100 g resin). In the available literature [33,42], epoxy composites have various contents of boric acid, often combined with a different filler. The choice of these quantities was justified by the work presented in [42]. On the other hand, the experiments presented in [32] contain an overview of information regarding the amounts of various antipyrene substances, including antiperspirants (phosphorus), which were used in amounts ranging from 0 to 1.4%. In the study presented by Demirham et al. [36], various amounts of boric acid (i.e., 1.25, 2.5, 3.75, and 5.0% by weight) were used as flame retardant additives. Visakh et al. [42] studied epoxy composites filled with boric acid and natural zeolite with different contents (1, 5, and 10% by weight). Murat Unlu et al. [55] used boron compounds in three concentrations: 1, 3, and 5% by weight.

The composition and designation of boron acid/epoxy composites are presented in Table 2. For comparative purposes, neat epoxy compounds without the addition of a modifier were also produced.

### 2.2. Preparation of Boron Acid/Epoxy Composites and Their Mechanical and Thermal Characterization

The preparation of the composite samples for mechanical tests and microscopic observations took place in several stages:

1. Preparation of cylindrical polymeric molds;
2. Weighing the ingredients;
3. Homogenization of boric acid in the epoxy resin;
4. Introduction of the curing agent;
5. Dispersing the curing agent in the modified epoxy resin;
6. Curing and post-curing processes;
7. Finishing the cured epoxy composite samples and conditioning them before any tests.

**Figure 2:**
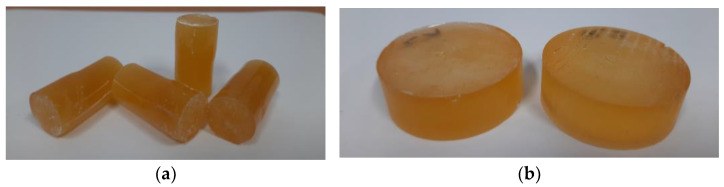
Samples of boron acid/epoxy resin composites: (**a**) type 1; (**b**) type 2.

To prepare the composite samples, two types of cylindrical molds were prepared. A silicone-based release agent Soudal (Soudal, Pionki, Poland) was applied to the inner surface of the mold using a spraying method from a distance of approximately 80–100 mm. The components of the composites were weighed using a laboratory balance (TM-1,5TN, FAWAG S.A, Lublin, Poland) with an accuracy of 0.1 g. The epoxy resin was weighed in a beaker with a capacity of 500 cm^3^ and the appropriate amount of boric acid was then added. The mixture underwent mechanical homogenization (mechanical disc mixer) for 3 min at a speed of 144 rpm. After this process, the appropriate amount of curing agent was added to the epoxy mixture. Each epoxy compound with the respective curing agent was mixed with a low-speed laboratory mixer for 3 min, deaerated, and poured into cylindrical molds (shown in Figure 2) with dimensions of 13.0 mm × 26.0 mm (Type 1—for mechanical tests) and 10.0 mm × 10.0 mm (Type 2—for microscopic observations), covered with a release agent. The composites were cured at laboratory temperature (21 ± 1 °C) and humidity (21 ± 2% R.H.) for 168 h, and then finished by milling. This treatment aimed at obtaining accurate dimensions (in the case of the mechanical test, 12.7 mm and 25.4 mm, according to the ASTM D695 standard (ASTM D695; Standard Test Method for Compressive Properties of Rigid Plastics. ASTM International, West Conshohocken, PA, USA, 2023) and perpendicularity deviations from the axis. The specimens were then conditioned at laboratory temperature (21 ± 1 °C) and humidity (21 ± 2% R.H.) before being tested. For comparison purposes, some epoxy specimens not containing boric acid were also produced.

A total of 48 composite samples were prepared for mechanical tests (6 samples for each composite formulation), and a total of 8 samples were prepared for thermal analyses (1 sample for each composite formulation).

After conditioning the epoxy composite samples, mechanical tests in compression mode in accordance with the ISO 604 standard (ISO 604; Plastics. Determination of compressive properties. International Organization for Standardization, Geneva, Switzerland, 2002) were carried out using a Zwick/Roell Z150 testing machine(Zwick/Roell GmbH&Co. KG, Ulm, Germany). The mechanical tests were executed using the following parameters: initial force of 200 N, test speed of 10 mm/min. The results of the mechanical tests were statistically analyzed using correlation and regression. The Pearson linear correlation coefficient r (X,Y) was adopted as a measure of the correlation of one variable (X) with the other variable (Y). Assumptions were made for the statistical analysis, and the corresponding statistical tests were used [57]. Statistical analysis was performed using the Statistica 13.1 program.

Thermal properties, in terms of glass transition temperature (Tg), were evaluated employing a differential scanning calorimetry (DSC1 Stare System) produced by Mettler Todedo (Columbus, OH, USA). The tests were carried out by scanning samples weighing approximately 10–20 mg and sealed in aluminum pans in an inert atmosphere (flow rate of nitrogen: 80 mL/min) between −15 °C and 150 °C. A constant heating rate of 10 °C/min was employed in each test. The average of the results of at least three experiments performed on each sample of epoxy composites was calculated.

The thermal degradation process as a function of the boric acid content in the epoxies was evaluated by thermogravimetric (TGA) analysis employing a thermogravimetric analyzer (TGA55) purchased by TA Instruments Company (Waters™, New Castle, DE, USA). This analysis was performed in the range of 25 °C–1000 °C, using a heating rate of 10 °C/min and nitrogen atmosphere (flow rate: 25 mL/min). The samples, weighing between 10 and 15 mg, were placed in an alumina crucible and tested. Three samples of each composition were analyzed and the results were averaged.

### 2.3. Flammability Tests

Flammability tests were performed based on the guidelines contained in the PN-EN 60695-11-10 standard (PN-EN 60695-11-10. Fire hazard testing—Part 11-10: Test flames—50 W horizontal and vertical flame test methods. Polish Committee for Standardization, Warsaw, Poland, 2014), Part 11-10. To this end, samples were placed horizontally on a universal stand used for testing the flammability of materials. The ignition source was a 50 W gas burner powered by methane. Comparative studies of changes in the temperature field in the area of the burning sample were carried out using a Flir A70 thermal imaging camera (FLIR Systems, Inc., Meer, Belgium). Thermal images were recorded from a distance of 700 mm with a thermal image recording frequency of 15 Hz. The recorded temperature ranged from 175 to 1000 °C. Images were recorded immediately after the ignition source was removed and after times t of 10, 20, 30, 40, 50, and 60 s. The analysis of the recorded thermal images (i.e., thermograms) was carried out using Flir Research Studio Player software v.1.7.

In the flammability test performed using method A, samples were mounted horizontally in the holder. Keeping the central axis of the burner tube inclined at an angle of 45° to the horizontal, the burner flame was applied to the lower edge of the free end of each sample in such a way that the flame covered it for a length of approximately 6 mm. The test flame was held for 10 ± 1 s without changing its position. After moving the burner away, the time meter was started and the time after which the flame front reached the marking made at a distance of 10 mm from the ignited end of the sample was recorded. The flame was extinguished when the time stopped.

The material was classified according to the criteria specified in the recommendations of the PN-EN 60695-11-10 standard.

## 3. Results

### 3.1. Mechanical Tests

#### 3.1.1. Compressive Strength

**Figure 3:**
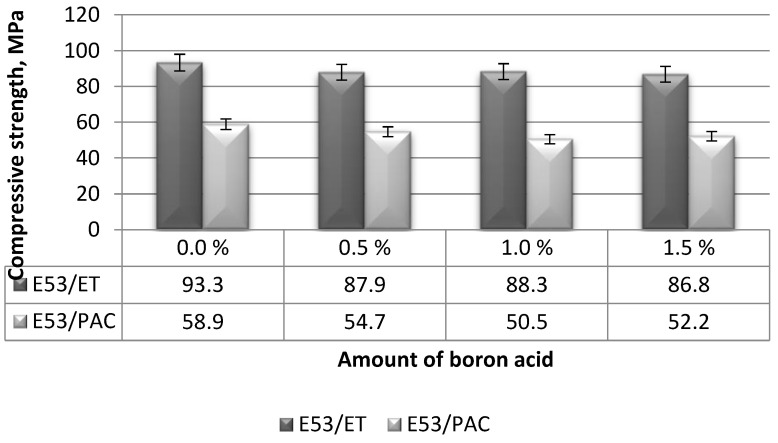
Compressive strengths calculated on the boron acid/epoxy resin composites.

The compressive strength values (average values from six samples) of the boron acid/epoxy resin composites are presented in Figure 3.

The compressive strengths of the boron acid/epoxy resin composites hardened by the amine curing agent (i.e., E53/ET specimens) were only slightly affected by the content of boric acid and ranged from 86.8 to 87.9 MPa (Figure 3). On the other hand, the highest compressive strength (93.3 MPa) was achieved by the neat (reference) epoxy samples (E53/ET), suggesting a limited negative effect of the addition of boric acid to this resin. In fact, the differences in compressive strength measured on the boron acid/epoxy resin composites and the reference epoxy compound are equal to the following:

−6.8% for the composite containing 0.5 g boric acid (E53/ET/H_3_BO_3_/0.5);
−6.3% for the composite with 1.0 g boric acid (E53/ET/H_3_BO_3_/1.0);
−8.2% for the composite with 1.5 g boric acid (E53/ET/H_3_BO_3_/1.5).

When analyzing the compressive strength results calculated for the boron acid/epoxy resin composites hardened by the polyamide curing agent, again reported in Figure 3, it was found that in this case, the highest compressive strength (equal to 58.9 MPa) was also achieved by the neat (reference) epoxy resin (i.e., E53/PAC). This value is 16.6% higher than the lowest compressive strength measured on the boron acid/epoxy resin composite containing 1.0 g of boric acid (i.e., E53/PAC/H_3_BO_3_/1.0 system), equal to 50.5 MPa. On the other hand, the highest compressive strength value (54.7 MPa) was achieved by the composite containing 0.5 g of boric acid (i.e., the E53/PAC/H_3_BO_3_/0.5 compound), this value being 7.1% lower than that measured on the neat epoxy resin. Therefore, even for this epoxy system, the addition of small quantities of boric acid has a limited effect on compressive strength.

In order to determine the relationship between the amount of boric acid and the compressive strength of the epoxy compounds, the method of linear correlation between two variables was used. The results are summarized in Table 3.

**Figure 4:**
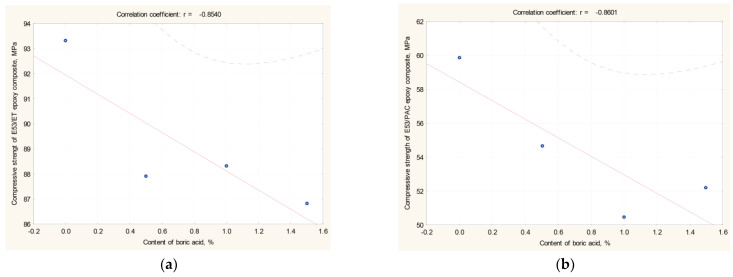
Correlation coefficient r (X, Y) of (**a**) E53/ET boron acid/epoxy resin composites; (**b**) E53/PAC boron acid/epoxy resin composites (results are reported in Table 3).

The correlation between the amount of boric acid and the compressive strength of boron acid/epoxy resin composites was determined using Pearson’s linear correlation coefficient r (X, Y), whose results are reported in Table 3 and Figure 4. If the value of the r coefficient is close to 1, then the examined variables X and Y are linearly related to each other. The other symbols reported in Table 3 represent the following: r^2^ represents the coefficient of determination; t represents the value of the t-statistic examining the significance of the correlation coefficient; and *p* calculates the significance level for the *t*-test.

Based on the results of the statistical analysis (reported in Table 3 and Figure 4) and the results of the relationship between compressive strength and the boric acid content in the epoxy composite, the following conclusions can be made:

(i) For epoxy composites cured using the amine curing agent:
  - The correlation coefficient (r) was found to be −0.854, which proves a strong linear relationship between the compressive strength of epoxy composites and the boric acid content in the composites;
  - The correlation coefficient is negative, which means that as the amount of boric acid in the composite increases, the strength decreases;
  - The coefficient of determination (r^2^) is 0.729, which means that almost 73% of the variation in the compressive strength can be attributed to the boric acid content in the epoxy composite.
(ii) For epoxy composites cured using the polyamide curing agent:
  - The correlation coefficient (r) was found to be −0.860, which again proves a strong linear relationship between the compressive strength of these epoxy composites and boric acid content;
  - The correlation coefficient is negative, which indicates that the strength decreases as the amount of boric acid in the composite increases;
  - There was a similar relationship as that in the case of the boron acid/epoxy resin composite hardened by amine curing agent.

It can therefore be concluded that the correlation between compressive strength and the content of boric acid in epoxy composites is not influenced by the type of curing agent employed in the resin, but rather by the amount of boron acid added.

#### 3.1.2. Compressive Modulus

**Figure 5:**
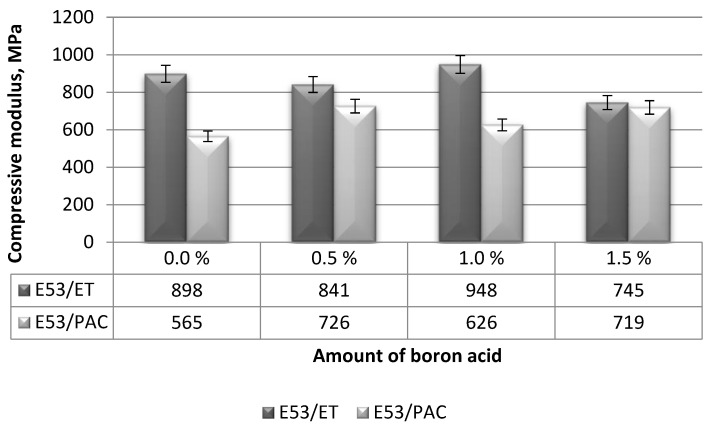
Compressive modulus measured on the boron acid/epoxy resin composites.

The compressive modulus values of the boron acid/epoxy resin composites are summarized in Figure 5.

The compressive modulus measured on the boron acid/epoxy resin composites cured using the amine curing agent (i.e., E53/ET samples) ranged between 745 MPa and 948 MPa, as reported in Figure 5. The highest compressive modulus (i.e., 948 MPa) was measured for the boron acid/epoxy resin composites containing 1.0 g boric acid (i.e., the E53/ET/H_3_BO_3_/1.0 formulation). This value is 5.5% greater than that measured on the reference sample (i.e., the neat epoxy resin). The lowest compressive modulus value was measured on the samples of the boron acid/epoxy resin composite containing 1.5 g boric acid (i.e., E53/ET/H_3_BO_3_/1.5) and it was 20.5% lower than that of the reference epoxy compound (i.e., E53/ET). Taking into account the ranges of variation in these results, it can be concluded that the addition of boric acid had a certain negative influence only in the case of the highest content of this additive.

Moving on to the analysis of the boron acid/epoxy resin composites cured using the polyamide curing agent, the lowest compressive modulus, equal to 565 MPa, was found for the neat epoxy resin (i.e., the E53/PAC compound). This value was 28.5% lower than the highest compressive modulus (i.e., 726 MPa) measured on the boron acid/epoxy resin composite containing 0.5 g of boric acid (i.e., the E53/PAC/H_3_BO_3_/0.5 formulation). For these epoxy composites, the values of compressive modulus increased as the boric acid content increased: the modulus of the epoxy composite containing 1.0 g of boric acid (i.e., E53/PAC/H_3_BO_3_/1.0) was 10.8% higher than that of the neat epoxy samples and that of the epoxy composite including 1.5 g of boric acid (i.e., E53/PAC/H_3_BO_3_/1.5) was 27.2% higher than that of the near epoxy. We conclude that, for this epoxy system, the inclusion of boric acid was beneficial to the compression modulus.

**Figure 6:**
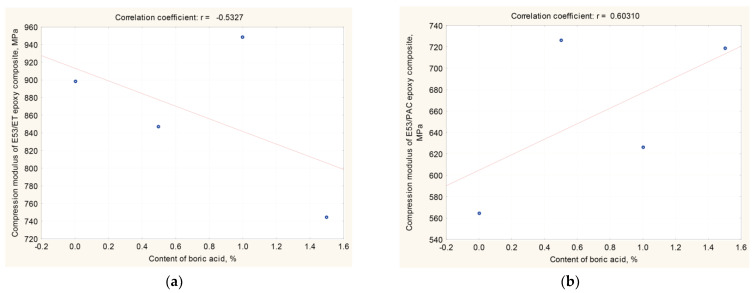
Correlation coefficient r (X, Y) of (**a**) E53/ET boron acid/epoxy resin composites; (**b**) E53/PAC boron acid/epoxy resin composites (results are reported in Table 4).

The compressive modulus results were also subjected to basic statistical analysis using the previously introduced coefficients, i.e., correlation and regression (reported in Table 4 and Figure 6).

Based on the results of the statistical analysis (reported in Table 4 and Figure 6) and the results of the relationship between the compressive modulus and boric acid content in the epoxy composite, the following observations can be made:

(i) For epoxy composites cured using the amine curing agent:
  - A correlation coefficient (r) equal to −0.533 proves a moderate linear relationship between the compressive modulus of the epoxy composites and boric acid content;
  - The correlation coefficient is found to be negative, indicating that the strength decreases as the amount of boric acid in the composite increases.
(ii) For epoxy composites cured using the polyamide curing agent:
  - A correlation coefficient (r) of 0.603 again indicates a moderate linear relationship between the compressive strength of this type of epoxy composite and the boric acid content;
  - In this case, the correlation coefficient is positive, which means that upon increasing the amount of boric acid in the composite, its compressive strength also increases;
  - There is an inverse relationship with respect to the boron acid/epoxy resin composites cured using the amine curing agent.

Based on the results of the performed statistical analysis, it can be concluded that the both the type of the curing agent and the amount of boric acid influence only to some extent the compressive modulus measured on the epoxy composites; in addition, the influence is not always detrimental.

#### 3.1.3. Compressive Strain

**Figure 7:**
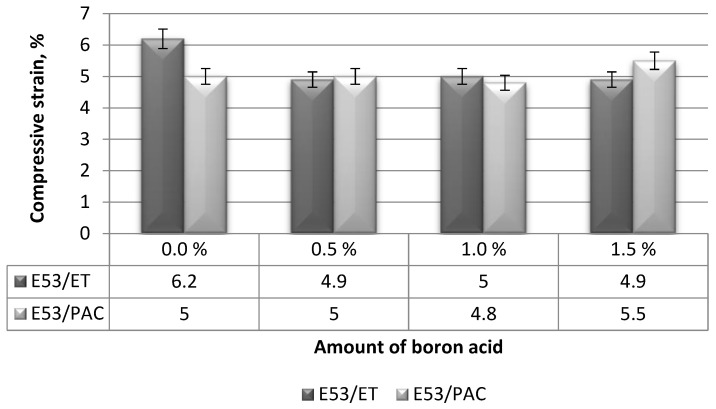
Compressive strain calculated on the boron acid/epoxy resin composites.

The strain values calculated on epoxy composites with different boric acid contents in compression mode are shown in Figure 7. For comparison purposes, the compressive strain of the reference epoxy resin is reported in the same Figure.

The compressive strain values of the boron acid/epoxy resin composites cured using the amine curing agent (i.e., E53/ET systems) were quite unaffected by the content of boric acid, ranging around 5% in all cases. The reference epoxy system (i.e., the E53/ET formulation) exhibits the highest compressive strain value, i.e., 6.2%, which is around 25% greater than that relative to all the boron acid/epoxy resin composites.

**Figure 8:**
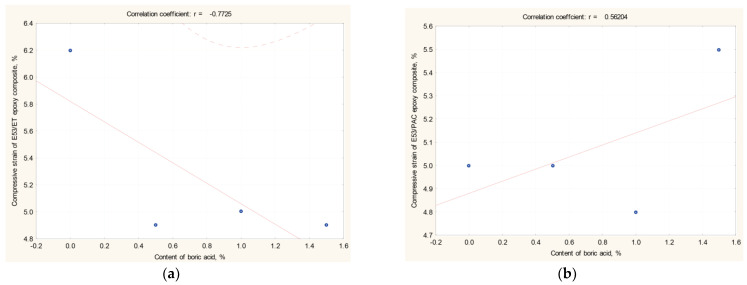
Correlation coefficient r (X, Y) of (**a**) E53/ET boron acid/epoxy resin composites; (**b**) E53/PAC boron acid/epoxy resin composites (Table 5).

Analyzing the results of the boron acid/epoxy resin composites hardened using the polyamide curing agent, the lowest compressive strain value (i.e., 4.8%) was measured on the composite containing 1.0 g boric acid (i.e., E53/PAC/H_3_BO_3_/1.0). On the other hand, the highest value of this characteristic, namely 5.5%, was measured on the epoxy composite containing 1.5 g boric acid (i.e., E53/PAC/H_3_BO_3_/1.5 compound). The compressive strain of both the reference epoxy resin and the boron acid/epoxy resin composite containing 0.5 g boric acid, i.e., the E53/PAC and E53/PAC/H_3_BO_3_/0.5 systems, respectively, was found to be 5%. The observed results suggest that there is no precise relationship between the compressive strain value and the boric acid content, and that the addition of boric acid has little influence on this characteristic. However, in order to confirm this hypothesis, the correlation and regression coefficients of these results were also determined; they are reported in Table 5 and Figure 8.

When analyzing the results of the basic statistical analysis presented in Table 5 and Figure 8, it is possible to observe that the relationship between compressive strain and the boric acid content in the epoxy composites is similar to what was previously observed for the modulus. In fact, the compressive strain measured on the epoxy composites cured using the amine curing agent decreases as the content of boric acid increases. On the other hand, the same property increases with increasing content of boric acid in the epoxy composites hardened by the polyamide curing agent. Therefore, a limited influence of the type of curing agent on the compressive characteristics of boric acid/epoxy resin composites is confirmed.

### 3.2. Thermal Analyses

#### 3.2.1. Glass Transition Temperature

Table 6 reports the average values of the glass transition temperature (Tg) measured on the epoxy composites that are the subject of the present study. The variation range of the experimental values was always less than 4%.

From the observation of the Tg values reported in Table 6, it can be concluded that the addition of boric acid to the epoxy systems did not significantly influence this temperature. Thus, boric acid does not act as a “plasticizer” for epoxy resin, regardless of the type of curing agent used. In fact, the differences in Tg observed in Table 6 are always lower than the experimental error measured in the calculations (i.e., less than 4%). Furthermore, these values do not have a precise trend as the additive content increases. For example, the highest Tg value found for E53/ET composites was measured for the system with the highest boric acid content (i.e., E53/ET/H_3_BO_3_/1.5) and it was almost equal to the Tg value measured for the neat resin cured with the same hardener, i.e., the E53/ET system. On the other hand, analyzing the compounds based on epoxy cured with polyaminoamide, the greatest (very similar) Tg values were measured for the composites containing 0.5 and 1.0 gr. of boric acid and the lowest for the system with the highest boric acid content (i.e., E53/PAC/H_3_BO_3_/1.5). As already underlined, however, these Tg variations fall within the range of variation of the results.

Finally, a comparison of the results reported in Table 6 allows us to conclude that using an amine-based hardener, it is possible to reach a significantly higher glass transition temperature value than the Tg achieved by the same styrene-modified epoxy resin cured with a polyamide, using the same curing cycle (in terms of temperature and time) for both systems. Therefore, the choice of the most appropriate curing agent for an epoxy resin must also be made based on the (maximum) service temperature expected for that specific application.

#### 3.2.2. Thermogravimetric Analysis

The thermal resistance of boric acid/epoxy composites in a non-oxidative atmosphere was analyzed using thermogravimetric (TGA) tests. In fact, from this analysis, it is possible to obtain useful indications on the effect of boric acid on the resistance of epoxy resin cured with two different hardeners to very high temperatures in the absence of oxygen. This information is very important for applications in which resin can reach high temperatures, even due to accidental causes. Thus, TGA was employed to evaluate the degradation range of temperatures during heating up to 1000 °C. The results of the TGA analysis are summarized in Table 7. In this table, the initial and final temperatures of the degradation process are indicated as “Onset Temperature” and “Endset Temperature”, respectively. Again, a very small range of variation of the experimental values (not exceeding 4%) was calculated.

No clear trend can be seen in the TGA results, regardless of the type of curing agent used in the styrene-modified epoxy resin systems. Indeed, the effect of boric acid is not very evident using this test: the beginning of the thermal degradation process, in the absence of oxygen, occurs more or less starting from around 350 °C. On the other hand, the temperature at the end of the degradation process seems to depend on the type of curing agent but not on the presence, and content, of boric acid, keeping in mind that the small temperature differences measured for the different compositions are still within the experimental error.

Not having obtained conclusive results on the effect of boric acid on the thermal resistance of styrene-modified epoxy resin from this test, we analyzed the flame behavior of epoxy systems depending on the boric acid content and the type of curing agent.

### 3.3. Flammability Tests

**Figure 9:**
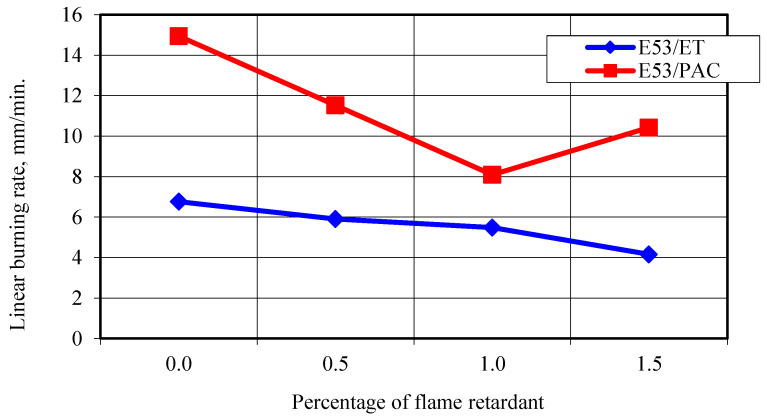
Linear burning rate depending on the content of the flame retardant (boron acid).

**Figure 10:**
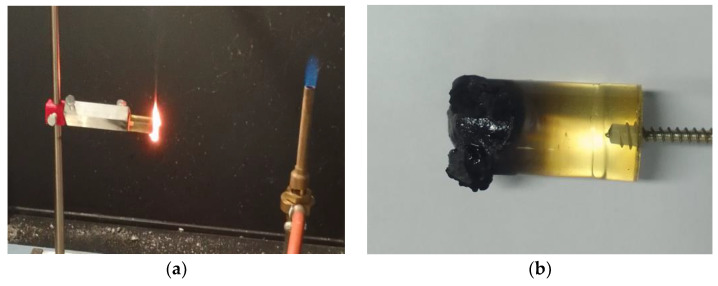
Views of E53/ET/H_3_BO_3_/1.0 sample of epoxy resin composites: (**a**) immediately after removing the flame source, (**b**) after combustion.

Table 8 and Figure 9 present the results of flammability tests performed in a horizontal arrangement using two series of four samples, each with different epoxy resin composites. Figure 10a shows an example of the appearance of a E53/ET/H_3_BO_3_/1.0 sample immediately after the removal of the flame source and the appearance of the same sample after combustion (Figure 10b).

In the case of the E53/ET epoxy resin composites, the introduction of an additional component caused a decrease in the linear burning rate of approximately 38% compared to the base value obtained for the unmodified material, which is a positive and significant effect. In general, the values of the linear burning rate obtained for the E53/PAC epoxy resin composites were much, even more than twice, higher than for the E53/ET epoxy resin composites, which indicates a deterioration in flammability caused by the base material or modifying agent used in this case. In the case of the E53/PAC epoxy resin composites, a significant reduction in the linear burning rate was achieved with the addition of 1.0% of the modifying agent, up to 8.09 mm/min, which indicates a reduction of 6.85 mm/min (approx. 69%) in the linear burning rate compared to the unmodified sample. With a modifier content of 1.5%, an increase in the linear burning rate to 10.43 mm/min was observed, the reason for which, however, is difficult to interpret unambiguously due to the small number of samples tested. Taking into account the values of the linear combustion rate obtained and the deviations from the standard requirements, all tested samples can be conventionally classified into the HB flammability class.

**Figure 11:**
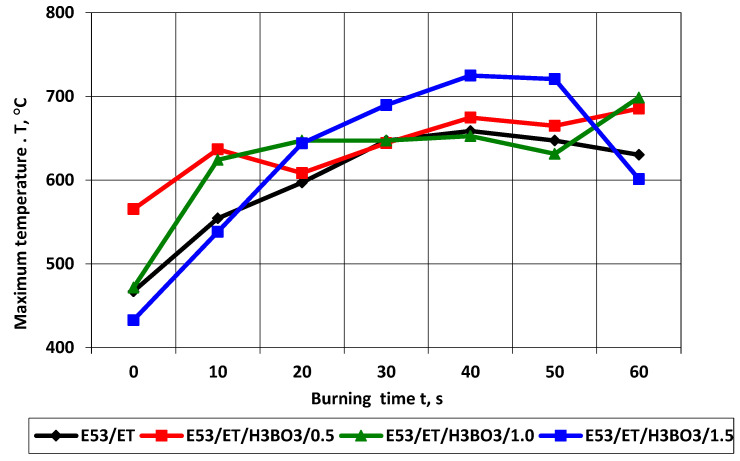
Maximum temperature in the burning area of E53/ET epoxy resin composite samples depending on burning time.

The analysis of the temperature field in the burning area of the samples in the horizontal burning test included determining the maximum temperature changes in the burning area, marked as “rectangle 1” in the thermal images, and the temperature along the burning sample in the area marked as “line 1”. The results of changes in the maximum temperature in the burning area of samples in series 1 are presented in Table 9 and Figure 11.

**Figure 12:**
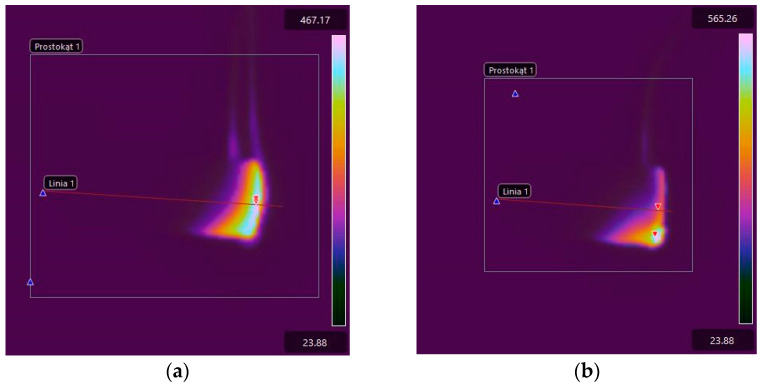

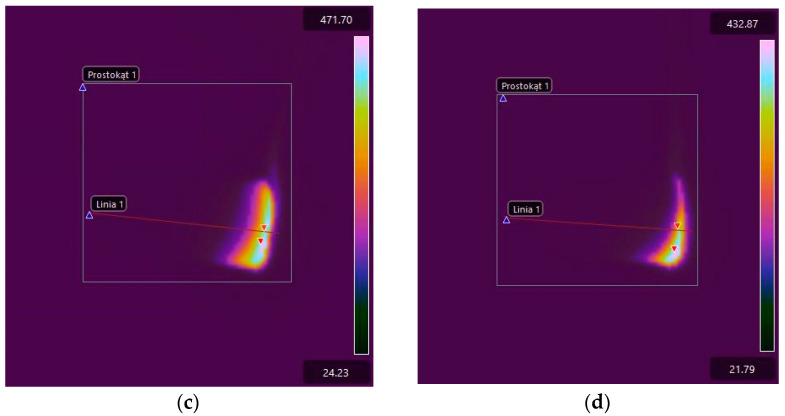
Thermal images of E53/ET epoxy resin composite samples recorded immediately after subtraction of the ignition source (time 0): (**a**) E53/ET; (**b**) E53/ET/H_3_BO_3_/0.5; (**c**) E53/ET/H_3_BO_3_/1.0; (**d**) E53/ET/H_3_BO_3_/1.5.

**Figure 13:**
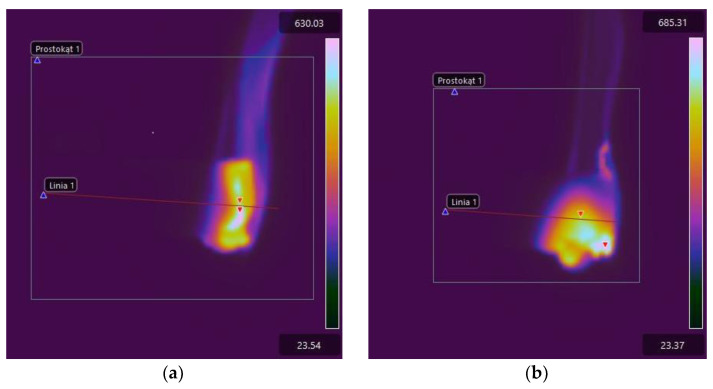

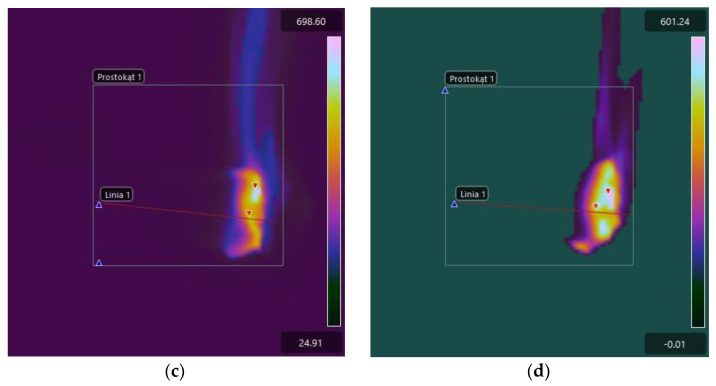
Thermal images of E53/ET epoxy resin composite samples recorded 60 s after ignition source subtraction: (**a**) E53/ET; (**b**) E53/ET/H_3_BO_3_/0.5; (**c**) E53/ET/H_3_BO_3_/1.0; (**d**) E53/ET/H_3_BO_3_/1.5.

The maximum temperature in the burning area of E53/ET epoxy resin composite samples generally increases with the burning time, which indicates the spread of the flame. In the case of sample E53/ET, the maximum temperature is slightly reduced after burning for more than 40 s, while for sample E53/ET/H_3_BO_3_/1.5, its reduction was observed after burning for 60 s. However, this may result from temporary disturbances in the combustion process and would require additional research. The highest temperature value in the burning area immediately after removing the ignition source (time 0) was recorded for the sample with 0.5 modifying agent content and was 565.26 °C; it was slightly lower at 1% modifying agent content (471.7 °C), amounted to 467.17 °C in the sample with no modification, and was the lowest for the sample with a modifying agent content of 1.5% (432.87 °C). In order to facilitate the evaluation of the obtained data, they are represented graphically in Figure 11. Figure 12 presents selected thermal images of the tested samples during burning, recorded immediately after subtracting the ignition source, for comparison purposes. The visible differences in the size of the area covered by combustion in individual thermal images correspond to the linear combustion rate decreasing with increasing amounts of the modifying agent; accordingly, this area was the largest in the sample without the modifier (Figure 12a). Similar relationships can be seen in Figure 13, which presents images recorded after 60 s of burning in the same order.

**Figure 14:**
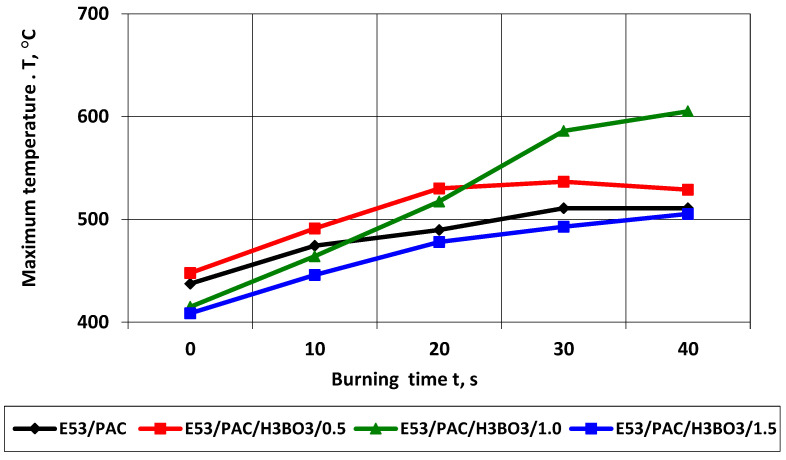
Maximum temperature in the burning area of E53/PAC epoxy resin composite samples depending on burning time.

Analogously to E53/ET epoxy resin composite samples, an analysis of the temperature field in the burning area in the horizontal burning test was carried out for E53/PAC epoxy resin composites. Due to the much higher burning rate in the case of samples of E53/ET epoxy resin composites, thermal images were only recorded up to a burning time of 40 s. The results of changes in the maximum temperature in the burning area of samples of this series are presented in Table 10 and Figure 14.

The temperature of E53/PAC epoxy resin composites also increased with the passage of burning time, which, similarly to E53/ET epoxy resin composites, indicated the spread of the flame. The combustion temperature in the case of samples of E53/PAC epoxy resin composites was generally lower than that of E53/ET epoxy resin composites, which is a favorable phenomenon for fire conditions. The highest temperature value in the burning area immediately after removing the ignition source (time 0) was also recorded in the case of the sample with 0.5% modifying agent content and was 447.89 °C; the lowest temperature was observed with 1.5% modifying agent content (408, 71 °C). In order to facilitate the evaluation of the obtained data, they are presented graphically in Figure 14. The maximum temperature in the burning area increases to a value slightly above 500 °C and then stabilizes with increasing burning time. Only in the case of the sample containing 1% of the modifying agent does the temperature rise above 600 °C.

**Figure 15:**
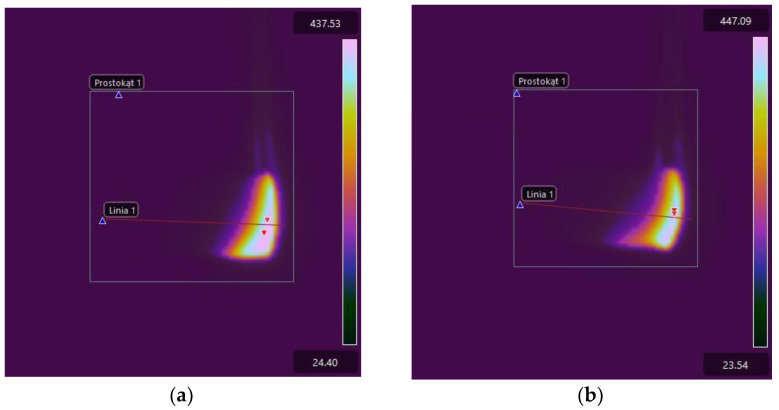

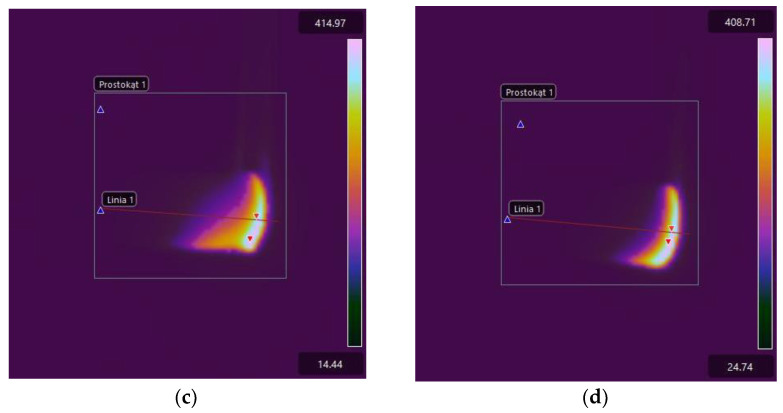
Thermal images of E53/PAC epoxy resin composite samples recorded immediately after the subtraction of the ignition source (time 0): (**a**) E53/PAC; (**b**) E53/PAC/H_3_BO_3_/0.5; (**c**) E53/PAC/H_3_BO_3_/1.0; (**d**) E53/PAC/H_3_BO_3_/1.5.

**Figure 16:**
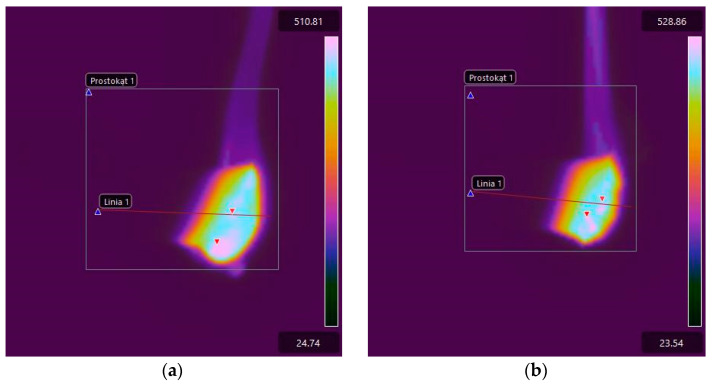

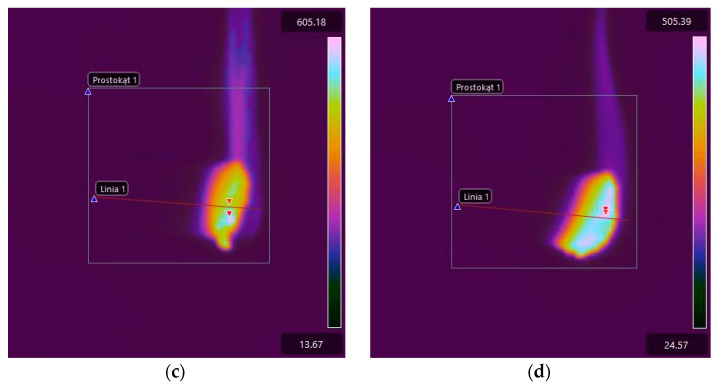
Thermal images of E53/PAC epoxy resin composite samples recorded 40 s after ignition source subtraction: (**a**) E53/PAC; (**b**) E53/PAC/H_3_BO_3_/0.5; (**c**) E53/PAC/H_3_BO_3_/1.0; (**d**) E53/PAC/H_3_BO_3_/1.5.

For comparison purposes, Figure 15 shows thermal images of the tested E53/PAC epoxy resin composite samples recorded immediately after subtracting the ignition source. The higher linear burning rate of samples of E53/PAC epoxy resin composites compared to E53/ET epoxy resin composites is reflected in the greater extent of the high-temperature area, especially on the lower surface of the samples. Combustion also occurs at a slightly lower temperature, which can be observed both in the thermograms shown in Figure 15 and those recorded after 40 s and shown in Figure 16.

## 4. Discussion

**Figure 17:**
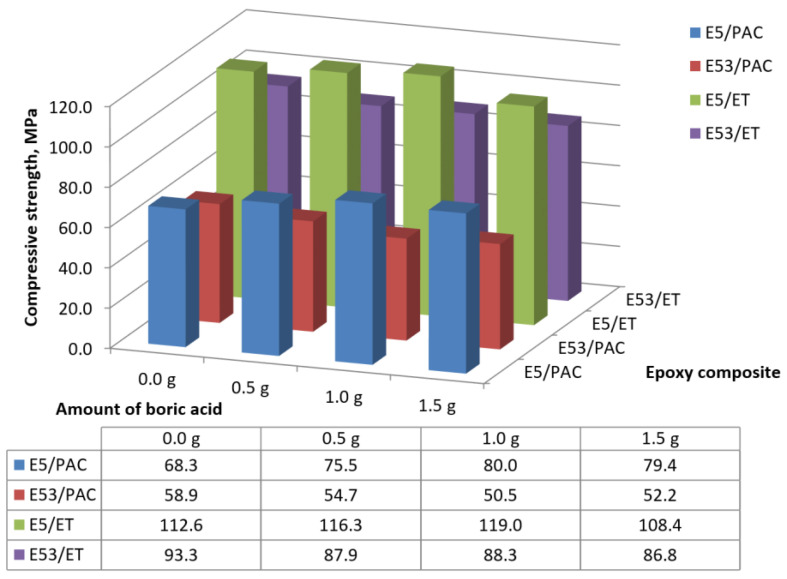
Average compressive strength calculated on boron acid/epoxy composites containing solvent-free resin (i.e., Epidian 5) and solvent-modified resin (i.e., Epidian 53), cured using amine or polyamide curing agents.

A discussion of the results of this study is presented below, while also taking into account the findings of a previous work, reported in [53], which discusses a solvent-free resin with an epoxy number of 0.48–0.51 mol/100 g. A comparison of the average compressive strength of epoxy composites containing different amounts of boric acid and based on an unmodified solvent-free (E5) resin or the solvent-modified (E53) resin, analyzed in this study, is presented in Figure 17. The same cross-linking agents were used to cure both epoxy resins.

The purpose of the comparison presented in Figure 17 is to highlight the effect of the type of epoxy resin constituting the matrix of the epoxy composites containing boron acid additive in different amounts. The average strength values are reported, not including the standard deviation of results to make the figures more readable. These data can be found in Section 3.1.1 and in the mentioned publication [58].

**Figure 18:**
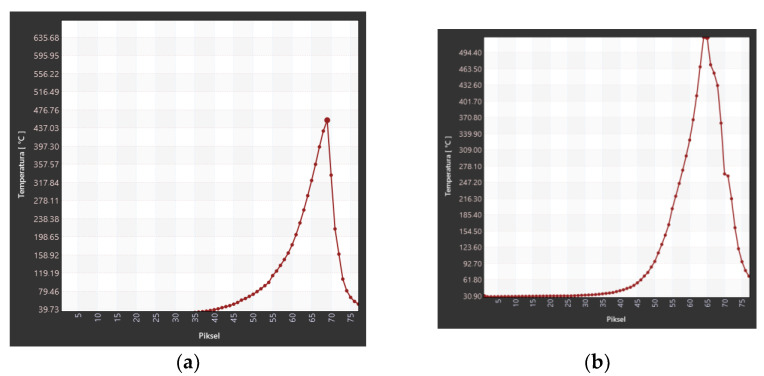
Temperature recorded along the axis of the E53/ET epoxy resin composite samples: (**a**) immediately after removing the ignition source; (**b**) 60 s after removing the ignition source.

From the analysis of the results presented in Figure 18, it is possible to conclude that epoxy compounds based on solvent-free epoxy resin (i.e., Epidian 5) are generally characterized by a higher compressive strength with respect to epoxy compounds based on solvent-modified epoxy resin (i.e., Epidian 53), irrespective of composition. Furthermore, boron acid/epoxy resin composites based on solvent-free epoxy resin display greater compressive strength than neat epoxy, while, in the case of composites produced with epoxy modified with styrene, the opposite trend is observed. The results of the calorimetric analysis carried out in the present study demonstrated, however, that boric acid does not act as a “plasticizer” for epoxy resin, since glass transition temperatures were not significantly modified in the presence of this additive. It must be underlined that the influence of an additive on the mechanical characteristics of an epoxy compound depends on many factors, starting from the type of matrix resin. As an example, Avada et al. [52] used boric acid to increase the mechanical properties of a composite based on polyvinyl alcohol (PVOH) and cellulose fibers; their results confirmed that an increase in mechanical strength was obtained in composites containing boric acid compared to those without this additive. On the other hand, Demirhan et al. [36] found a slight decrease in tensile strength and an increase in bending strength in polypropylene–MMT (i.e., montmorillonite) composites containing various amounts of boric acid (from 1.25 to 5.0 wt.%).

**Figure 19:**
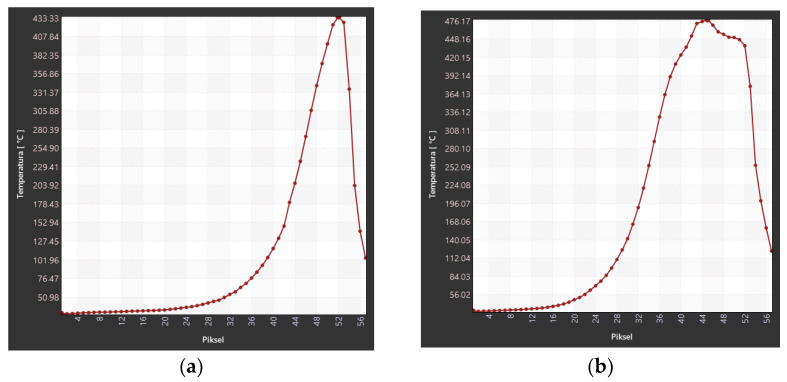
Temperature recorded along the axis of the E53/PAC epoxy resin composite sample: (**a**) immediately after removing the ignition source; (**b**) 40 s after removing the ignition source.

Referring to the type of the curing agent used to cure the epoxies, the composites cured using the amine curing agent display higher compressive strength values than those cured using the polyamide, irrespective of the kind of epoxy resin. Moreover, based on the data reported in Figure 19, it can be concluded that, in most cases, the greatest differences in compressive strength values are observed for epoxy composites cured using a polyamide curing agent with an amine number of 290–360 mg KOH/g with respect to composites hardened by an amine curing agent with an amine number of 700–900 mg KOH/g. In the case of boron acid/epoxy resin composites (matrix: Epidian 53 epoxy resin), the differences in compressive strength, depending on the type of the curing agent, were about 40% (37.8–42.8%), as reported in Figure 18. On the other hand, a comparison of the Tg values measured on the different epoxy systems analyzed in the present study (reported in Table 6) demonstrated the superiority of styrene-modified epoxy resin cured with an amine-based curing agent over that hardened using a polyamide.

Based on the obtained results, it is confirmed that the selection of the curing agent is very important, especially when choosing the formulation of an epoxy composite to be applied in the construction field. The type of curing agent can influence the properties and characteristics of epoxy composites due to its chemical composition and structure, which are capable of giving rise, for example, to more or less rigid cross-linked structures. Furthermore, the choice of the most suitable curing agent also depends on the curing temperature. For example, aliphatic amines are suitable for curing at room temperature, while aromatic ones require high curing temperatures. Finally, the choice of hardener is also a function of the final application intended for the resin. In this regard, Saeedi et al. [13] investigated the effect of curing mechanisms on selected electrical parameters of a commercial DGEBA resin (diglycidyl ether of bisphenol-A based epoxy) cured with amine and anhydride curing agents. These authors reported that both the formation of a more or less dense network and the reactivity of the DGEBA resin depend on the curing agent used. Ignatenko et al. [21] studied the curing reactions of a DGEBA with a mixture of two curing agents. They confirmed that the properties of cured resins depend on both the curing agent employed and on the curing conditions. In relation to the results observed in the present study, it is known that polyamide curing agents, compared to amines, provide greater flexibility to cured epoxy resins at the expense of other mechanical characteristics [59].

Both epoxy resins can be modified by introducing various types of additives, including other polymers [60,61], and both resins are intended, among others, for coating applications and as sealing adhesives; however, they have different viscosities, which may be important in some applications. Generally, the use of Epidian 5 epoxy resin allows for the creation of high-viscosity compositions, while the use of Epidian 53 epoxy resin creates low-viscosity compositions (although this also depends on the type of curing agent). An unmodified, solvent-free resin with an epoxy number of 0.48–0.51 mol/100 g has a viscosity in the range of 2000–3000 mPa·s, while the viscosity of a solvent-modified epoxy resin is in the range of 900–1500 mPa·s.

The advantage of the amine-cured epoxy resin composition used is that it can be used as a polymer coating. Nowadays, such coatings are increasingly used on floors and walls in various buildings. The advantage of the composition of epoxy resins with a polyamide curing agent is that they are used to join elements exposed to deformation, because this curing agent increases the elasticity and impact strength of the composition. At the same time, a negative feature of these compositions is that, depending on the amount of the polyamide curing agent, they are less hard and less resistant to increased temperatures.

When analyzing the results obtained in the flammability test, the temperature along the sample axis was compared for both analyzed groups of epoxy composites (Figure 18 and Figure 19).

The changes in temperature measured along the longitudinal axis of the sample (Figure 18) confirm the increase in combustion temperature and the expansion of the high-temperature area, which proves that the sample surface covered by the flame increases with the passage of combustion time.

In order to compare the temperature changes in unmodified E53/PAC epoxy resin composites (Figure 19) with those in the analogous samples of E53/ET epoxy resin composites (Figure 18) measured on sample z along its longitudinal axis, the appropriate charts are presented in Figure 19. In these charts, one can notice a much larger surface area covered by combustion on the E53/PAC epoxy resin composite sample, which confirms the higher linear combustion rate, with lower temperature values than those obtained for the E53/ET epoxy resin composites.

Based on the conducted tests, it was determined that the values of the linear burning rate obtained for E53/PAC epoxy resin composites were significantly, even more than twice, higher than those obtained for E53/ET epoxy resin composites, which proves that the base material of E53/PAC epoxy resin composites is more susceptible to combustion. At the same time, a beneficial effect of the impact of the applied materials was observed for both tested materials. The use of the modifying agent manifested in a decrease in the linear combustion rate with increases in its content. With a modifier content of 1.5%, an increase in the linear combustion rate was observed, but the reason for this is difficult to determine. In turn, Hergenrother et al. [62] investigated flame retardant epoxy resins containing phosphorus and several formulations, which showed excellent flame retardancy with low phosphorus content as low as 1.5% by weight. Hamciuc et al. [25] showed that the introduction of 2 wt. boron and 2% by weight phosphorus significantly reduced the flammability of the thermosetting epoxy. For this sample, THR and HRC decreased significantly, while the amount of residue increased significantly. Compared to pure EP-0, MCC results showed that the values of PHRR, THR, and HRC decreased by 55.03, 22.93, and 58.365%, respectively. The authors [25] demonstrated that the fire resistance of epoxy resin was significantly improved by the simultaneous introduction of DOPO (9,10-Dihydro-oxa-10-phosphophenanthrene-10-oxide) and H_3_BO_3_ derivatives. Kumar et al. [34] also reported that flammability decreased with the addition of flame retardant additives, comparing WPC composites containing APP 10%/Ba-Bx 5% and pure composites. In turn, Murat Unlu et al. [55] investigated the effect of three different boron compounds (BA, ZB, and MB) on the fire retardant properties of an APP-based intumescent coating. According to the TGA results, the addition of boron compounds increased the decarburization efficiency, with the most significant increase achieved after the addition of ZB. The authors showed that as the amount of added boron compounds increases, the fire retardant properties of the intumescent coating decrease due to the reduction in the height of the intumescent char.

Taking into account the obtained values of the linear burning rate and the deviations from the standard requirements, all tested samples can be classified into the HB flammability class, which, however, would require the application of the measurement conditions specified in the standard. The maximum temperature in the burning area of E53/ET epoxy resin composite samples generally increases with burning time, which indicates that the fire flares up and is an unfavorable phenomenon. The introduction of modifiers also results in an increase in the maximum temperature in the burning area of samples from this series. The maximum temperature in the burning area of samples of E53/PAC epoxy resin composites also increases with the passage of burning time, but it is lower than that of E53/ET epoxy resin composites, which is a favorable phenomenon for fire conditions. It should be noted, however, that in terms of fire hazards, a greater rate of fire spread, defined in the reported studies as a greater linear combustion rate, poses a greater threat. The recorded thermal images confirm the relationships obtained in the measurements, enabling the assessment of the extent and spread of the burning process.

## 5. Conclusions

In the present study, the effect of the inclusion of boric acid (used as a flame retardant) in a styrene-modified epoxy resin on its thermal and compressive mechanical properties was investigated. From the analysis of the obtained results, and taking into account the current literature, the following conclusions can be drawn:

- First, analyzing the effect of the type of curing agent, amine-cured epoxy composites exhibit higher compressive strength values and greater glass transition temperatures than polyamide-cured ones, regardless of the type of epoxy resin;
- Higher glass transition temperatures using the amine curing agent are also achieved when boric acid is added to the epoxy resin. The only exception is represented by the epoxy composite containing the highest boric acid content;
- The results of mechanical tests in compression mode do not indicate a clear trend of the influence of boric acid content on the mechanical characteristics of the tested epoxy composites. Similarly, even the Tg values, calculated by DSC analysis, appear to be unaffected by the presence and content of this additive. From an economic point of view, therefore, lower boric acid content could be used to obtain the same characteristics at a lower cost for raw materials;
- Since the results of the thermogravimetric (TGA) tests did not provide conclusive results on the effect of the presence of boric acid on the thermal degradation of the resin, the flame behavior of the epoxy systems under analysis was analyzed;
- The values of the linear burning rate obtained for the epoxy resin composites cured using the polyamide agent were significantly greater (i.e., more than twice as high) than those measured on epoxy resin composites cured using an amine, which proves that the epoxy system cured using a polyamide is more susceptible to combustion;
- A beneficial effect of the addition of boric acid on epoxy composites has been observed, manifested in a decrease in the linear burning rate as the content of this compound increases;
- The maximum temperature in the combustion area of polyamide-cured epoxy resin composites increases with burning time, but it remains lower than that measured for amine-cured epoxy resin composites; this represents an advantage in the event of fire.

As a future perspective, epoxy composites containing boric acid and cured using an amine curing agent could be tested as floor lining. On the other hand, composites based on epoxy resins and boric acid hardened using a polyamide could be tested as adhesives for joining construction materials.

## Figures and Tables

**Table 1:** Physicochemical properties of the curing agents used (Sarzyna Resins, Nowa Sarzyna, Poland).

Properties	Curing Agent Type	
	Amine	Polyamide
	Adduct of Aliphatic Amine (Triethylenetetramine) and Aromatic Glycidyl Ether	Polyaminoamide
	Trade Name	
	ET	PAC
Amine number [mg KOH/g]	700–900	290–360
Viscosity 25 °C [m·Pas]	200–300	10,000–25,000
Density at 20 °C [g/cm^3^]	1.02–1.05	1.10–1.20
Stoichiometric ratio: epoxy resin/curing agent	100:18	100:80

**Table 2:** Boron acid/epoxy composites and relative neat epoxy resin investigated in the present study.

Resin	Curing Agent	Boric Acid (H_3_BO_3_) Content (%/per 100 g Resin)	Denotation
Epoxy resin average molecular weight ≤ 700 (Epidian 53)	Amine (ET)	0.5	E53/ET/H_3_BO_3_/0.5
		1.0	E53/ET/H_3_BO_3_/1.0
		1.5	E53/ET/H_3_BO_3_/1.5
		0.0	E53/ET
	Polyamide (PAC)	0.5	E53/PAC/H_3_BO_3_/0.5
		1.0	E53/PAC/H_3_BO_3_/1.0
		1.5	E53/PAC/H_3_BO_3_/1.5
		0.0	E53/PAC

**Table 3:** Correlation between the amount of boric acid and the compressive strength measured on the boron acid/epoxy resin composites.

X Variable	Amount of Boric Acid	
Y Variable	Compressive Strength of Boron Acid/Epoxy Resin Composites	
	Cured Using Amine Curing Agent (Base: E53/ET)	Cured Using Polyamide Curing Agent (Base: E53/PAC)
r (X, Y)	−0.854	−0.860
r^2^	0.729	0.740
t	−2.321	−2.385
*p*	0.014	0.014
Regression coefficient X to Y	−3.820	−5.460
Regression coefficient Y to X	−0.191	−0.136

**Table 4:** Correlation between the amount of boric acid and the compressive modulus measured on the boron acid/epoxy resin composites.

X Variable	Amount of Boric Acid	
Y Variable	Compressive Modulus of Boron Acid/Epoxy Resin Composites	
	Cured Using Amine Curing Agent (Base: E5/ET)	Cured Using Polyamide Curing Agent (Base: E5/IDA)
R (X, Y)	−0.533	0.603
r^2^	0.284	0.364
t	−0.890	1.069
*p*	0.046	0.039
Regression coefficient X to Y	7.160	7.240
Regression coefficient Y to X	−0.004	0.005

**Table 5:** Correlation between the amount of boric acid and the compressive strain measured on boron acid/epoxy resin composites.

X Variable	Amount of Boric Acid	
Y Variable	Compressive Strain of Boron Acid/Epoxy Resin Composites	
	Cured Using Amine Curing Agent (Base: E5/ET)	Cured Using Polyamide Curing Agent (Base: E5/IDA)
r (X, Y)	−0.772	0.562
r^2^	0.597	0.316
t	−1.720	0.961
*p*	0.046	0.043
Regression coefficient X to Y	−0.760	0.260
Regression coefficient Y to X	−0.785	1.215

**Table 6:** Average values of the glass transition temperature (Tg) calculated on boron acid/epoxy composites and relative neat epoxy compounds.

System	Glass Transition Temperature (Tg) [°C]
E53/ET E53/ET/H_3_BO_3_/0.5 E53/ET/H_3_BO_3_/1.0 E53/ET/H_3_BO_3_/1.5 E53/PAC E53/PAC/H_3_BO_3_/0.5 E53/PAC/H_3_BO_3_/1.0 E53/PAC/H_3_BO_3_/1.5	60.8
	58.6
	59.8
	60.6
	38.6
	39.9
	38.5
	40.7

**Table 7:** Average results of thermogravimetric analysis (TGA) performed on boron acid/epoxy composites and relative neat epoxy compounds.

System	Onset Temperature [°C]	Endset Temperature [°C]
E53/ET E53/ET/H_3_BO_3_/0.5 E53/ET/H_3_BO_3_/1.0 E53/ET/H_3_BO_3_/1.5 E53/PAC E53/PAC/H_3_BO_3_/0.5 E53/PAC/H_3_BO_3_/1.0 E53/PAC/H_3_BO_3_/1.5	347.8	408.8
	349.3	412.9
	349.7	410.5
	341.3	407.8
	351.7	453.2
	348.6	452.0
	349.2	452.8
	347.5	452.3

**Table 8:** Results of flammability tests in a horizontal arrangement.

Sample Designation of Epoxy Resin Composites	Length of Sample Failure L [mm]	Burning Time of the Measurement Section t_1_ [s]	Linear Burning Rate v [mm/min]	Flammability Class According to Horizontal Test
E53/ET				
E53/ET/H_3_BO_3_/0.5	11.61	103	6.76	HB
E53/ET/H_3_BO_3_/1.0	9.04	92	5.90	HB
E53/ET/H_3_BO_3_/1.5	8.13	89	5.48	HB
E53/ET	8.53	123	4.16	HB
E53/PAC				
E53/PAC/H_3_BO_3_/0.5	12.95	52	14.94	HB
E53/PAC/H_3_BO_3_/1.0	9.79	51	11.52	HB
E53/PAC/H_3_BO_3_/1.5	9.44	70	8.09	HB
E53/PAC	9.21	53	10.43	HB

**Table 9:** Results of maximum temperature tests in the burning area for E53/ET epoxy resin composites.

Burning Time t [s]	Maximum Temperature in the Burning Area T [°C]			
	E53/ET/H_3_BO_3_/0.5	E53/ET/H_3_BO_3_/1.0	E53/ET/H_3_BO_3_/1.5	E53/ET
0	467.17	565.26	471.70	432.87
10	554.30	636.54	624.01	538.27
20	597.03	608.45	647.21	643.74
30	646.99	644.10	647.18	689.55
40	658.57	674.56	652.48	724.78
50	647.21	664.77	631.39	720.64
60	630.03	685.31	698.60	601.24

**Table 10:** Results of maximum temperature tests in the burning area for E53/PAC epoxy resin composites.

Burning Time t [s]	Maximum Temperature in the Burning Area T [°C]			
	E53/PAC/H_3_BO_3_/0.5	E53/PAC/H_3_BO_3_/1.0	E53/PAC/H_3_BO_3_/1.5	E53/PAC
0	437.53	447.89	414.97	408.71
10	474.46	491.19	464.18	446.00
20	489.77	530.04	517.38	478.10
30	510.90	536.67	586.11	492.86
40	510.81	528.86	605.18	505.39
50	437.53	447.89	414.97	408.71
60	474.46	491.19	464.18	446.00

## Data Availability

The data supporting the findings of this study are available from the corresponding author upon reasonable request.
